# Preoperative biopsy underestimates tumour budding but retains prognostic value in oral squamous cell carcinoma

**DOI:** 10.3389/fonc.2026.1932265

**Published:** 2026-09-30

**Authors:** Janik Riese, Claudio Cacchi, Julia Peppler, Guillaume Bonni, Julia Pannhausen, Anna Klein, Flurin Müller-Diesing, Danny Jonigk, Frank Hölzle, Maurice Klein

**Affiliations:** 1 Institute of Pathology, University Hospital, Rheinisch Westfälische Technische Hochschule (RWTH) Aachen University, Aachen, Germany; 2 Center for Integrated Oncology (CIO), CIO Aachen Bonn Cologne Duesseldorf (ABCD), Aachen, Germany; 3 Department of Oral and Maxillofacial Surgery, University Hospital, Rheinisch Westfälische Technische Hochschule (RWTH) Aachen University, Aachen, Germany; 4 Department of Otorhinolaryngology, School of Medicine, University Hospital Aachen, Aachen, Germany; 5 German Center for Lung Research, Deutsches Zentrum für Lungenforschung (DZL), Biomedical Research in Endstage and Obstructive Lung Disease (BREATH), Hanover, Germany

**Keywords:** biomarker, cancer, oral squamous cell carcinoma (OSCC), preoperative biopsy, risk stratification, tumour budding, tumour marker

## Abstract

**Introduction:**

Tumour budding is an established prognostic marker in oral squamous cell carcinoma (OSCC), but its reliability and prognostic performance in preoperative biopsy specimens remain uncertain. This study compared tumour budding assessment in matched biopsy and resection specimens and evaluated their respective prognostic value.

**Methods:**

We retrospectively analysed 256 patients with OSCC and matched preoperative biopsy and resection specimens. Tumour budding at the invasive front was assessed on hematoxylin and eosin-stained sections according to the International Tumour Budding Consensus Conference criteria and agreement between biopsy- and resection-based assessment was evaluated alongside associations with clinicopathological characteristics, disease-specific survival and occult nodal metastasis using logistic regression and Cox proportional hazards models.

**Results:**

Biopsy-derived tumour budding showed only weak correlation with resection findings and systematically underestimated high tumour budding, resulting in poor categorical agreement (κ = 0.11) and low sensitivity (9.8%) despite high specificity (98.9%). Nevertheless, both biopsy- and resection-based tumour budding were associated with disease-specific survival when mutually adjusted for each other. The C-index was numerically higher for resection-based than biopsy-based assessment (0.72 vs. 0.60), with only a modest increase when both measures were combined (C-index 0.74). Both biopsy- and resection-derived tumour budding were also associated with local recurrence and distant metastasis in mutually adjusted Cox models, although these secondary analyses remain exploratory. Interpretation is limited by the small biopsy high-budding group (n = 10) and the limited number of events for several secondary outcomes. For later lymph-node metastasis, only resection-derived tumour budding remained statistically significant after mutual adjustment, whereas the association with biopsy-derived tumour budding was borderline. Resection-derived tumour budding, but not biopsy-derived tumour budding, remained associated with occult nodal metastasis after mutual adjustment, while no statistically significant specimen-type interactions were detected for the evaluated clinicopathological characteristics, with several estimates remaining imprecise.

**Discussion:**

Preoperative biopsy-derived tumour budding may provide clinically relevant prognostic information in OSCC despite systematically underestimating high tumour budding. Although resection-based assessment showed a numerically higher C-index, biopsy assessment may provide complementary preoperative prognostic information but should not be considered a surrogate for resection-based assessment.

## 1 Introduction

Oral squamous cell carcinoma (OSCC) is the most common malignancy of the oral cavity and remains associated with substantial morbidity and mortality despite advances in surgical techniques, reconstruction and multimodal therapy (1). Although the TNM classification remains the cornerstone of prognostic assessment, patients with similar clinicopathological stage frequently experience markedly different clinical outcomes (2, 3). Consequently, considerable effort has been directed towards identifying histopathological biomarkers that more accurately reflect tumour aggressiveness and improve individualized risk stratification (3–5).

Tumour budding, defined as isolated single tumour cells or clusters of up to four tumour cells at the invasive tumour front, has emerged as one of the most promising prognostic biomarkers in epithelial malignancies (6, 7). Originally established in colorectal cancer, tumour budding has subsequently been recognized as a marker of epithelial–mesenchymal transition, local invasiveness and metastatic potential (8–11). In OSCC, numerous retrospective studies and several systematic reviews and meta-analyses have consistently demonstrated that high tumour budding is associated with adverse clinicopathological features, cervical lymph node metastasis, disease recurrence and reduced survival, supporting its potential incorporation into routine pathological reporting (12–14). Recent studies have further suggested that assessment across the entire invasive front may improve reproducibility and prognostic performance compared with conventional hotspot-based evaluation (15, 16). The clinical relevance of tumour budding has recently been further emphasized by the 9^th^ edition of the UICC TNM Classification of Malignant Tumours, in which tumour budding is listed among additional tumour-related prognostic factors (17). However, no detailed methodological guidance is provided regarding its standardized assessment, particularly with respect to the type of specimen used for evaluation.

Despite this growing evidence, tumour budding is almost exclusively assessed in surgical resection specimens. However, many important clinical decisions, including the extent of surgery, management of the clinically negative neck and the consideration of neoadjuvant treatment strategies, must be made before definitive tumour resection. Consequently, there is increasing interest in evaluating whether tumour budding can be reliably assessed in preoperative biopsy specimens. Previous investigations in colorectal carcinoma and pulmonary squamous cell carcinoma have demonstrated that biopsy-derived tumour budding retains prognostic value but may underestimate tumour budding owing to limited sampling of the invasive front (18–20). Similarly, biopsy-based tumour budding has shown promising prognostic value in OSCC, although direct comparisons with matched resection specimens remain scarce and the degree of concordance between both specimen types has not been comprehensively characterized (21).

A fundamental unanswered question is therefore whether tumour budding assessed in diagnostic biopsies can serve as a reliable surrogate for resection-based assessment or whether both specimen types capture distinct prognostic information. Therefore, the aim of the present study was to compare tumour budding assessed in matched preoperative biopsy and surgical resection specimens from patients with OSCC. Specifically, we evaluated the concordance between both sampling modalities, determined their respective associations with clinicopathological characteristics, disease-specific survival (DSS) and occult cervical lymph node metastasis and investigated whether biopsy- and resection-derived tumour budding provide overlapping or incremental prognostic information.

## 2 Materials and methods

### 2.1 Patients and clinical data

The present study included patients older than 18 years with histologically confirmed oral squamous cell carcinoma (OSCC) diagnosed between 2019 and 2023. Only cases with matched biopsy and resection specimens were included in the analysis. Biopsy specimens from the surgery department are routinely taken at the border of the tumour to the surrounding tissue. Only biopsy–resection pairs with clear transition of cancer to surrounding tissue were analysed to ensure quantification at the peritumoural invasion front. Patients with a second carcinoma within the head and neck squamous cell carcinoma (HNSCC) spectrum or with recurrent disease were also included. The frequency of these cases and relevant clinicopathological characteristics are summarized in **Table 1**. These patients were retained in the analyses according to the predefined study cohort, with the OSCC under investigation considered the index tumour. Tumour sites comprised the floor of the mouth, tongue, buccal mucosa, gingiva of upper and lower jaw as well as lesions involving multiple regions. Oropharyngeal carcinomas were excluded. Postoperative and follow-up data, along with tumour characteristics, were retrospectively extracted from the institutional hospital information system. The following clinicopathological parameters were collected: sex, age at diagnosis, tumour location and extent, presence of a second HNSCC, TNM classification including grading, resection margin status, lymphatic, venous and perineural invasion, clinical lymph node status, extranodal extension of lymph node metastases, tumour size and depth of invasion. Clinical nodal status was determined during initial staging. Patients were classified as cN0 when there was no clinical and radiological evidence of cervical lymph-node metastasis before primary treatment. The analysis of occult lymph-node metastasis was restricted to patients classified as cN0 who underwent neck dissection as part of their primary treatment and for whom pathological nodal status was available. All neck dissections included in this analysis were performed at our institution; therefore, no cN0 patients were excluded because neck dissection had been performed elsewhere. Occult nodal metastasis was defined as histopathologically confirmed cervical lymph-node metastasis in a patient classified as cN0 before surgery. Follow-up data included duration of follow-up, local recurrence, later lymph node metastases, distant metastases during follow-up, overall survival and disease-specific survival. Later lymph-node metastasis was defined as cervical nodal metastatic disease diagnosed during follow-up after completion of primary treatment and was analysed separately from occult nodal metastasis identified at primary neck dissection.

**Table 1:** Clinicopathological characteristics of the study cohort.

Parameters	Biopsy TB_low_	Biopsy TB_high_	Resection TB_low_	Resection TB_high_
Age [years]^#^	66.0 (17.0)	64.5 (11.5)	65.0 (16.75)	66.0 (16.5)
Gender^Δ^				
*Female* *Male*	128 (52%) 118 (48%)	2 (20%) 8 (80%)	94 (54%) 80 (46%)	36 (44%) 46 (56%)
Tumor localisation^Δ^				
*Floor of mouth* *Oral tongue* *Buccal mucosa* *Mandibular gingiva* *Maxillary gingiva* *Overlapping*	56 (22.8%) 76 (30.9%) 23 (9.3%) 52 (21.1%) 27 (11.0%) 11 (4.5%)	1 (10%) 3 (30%) 1 (10%) 3 (30%) 1 (10%) 1 (10%)	41 (23.6%) 64 (36.8%) 17 (9.8%) 33 (19%) 12 (6.9%) 6 (3.4%)	16 (19.5%) 15 (18.3%) 7 (8.5%) 22 (26.8%) 16 (19.5%) 6 (7.3%)
Tumor stage^Δ^				
*pT1* *pT2* *pT3* *pT4*	89 (36.2%) 56 (22.8%) 41 (16.7%) 60 (24.4%)	1 (10%) 3 (30%) 1 (10%) 5 (50%)	84 (48.3%) 33 (19.0%) 24 (13.8%) 33 (19.0%)	6 (7.3%) 26 (31.7%) 18 (22.0%) 32 (39.0%)
Nodal stage^Δ^				
*pN0* *pN1* *pN2* *pN3*	168 (70.3%) 26 (10.9%) 19 (7.9%) 26 (10.9%)	6 (60%) 1 (10%) 1 (10%) 2 (20%)	139 (82.2%) 14 (8.3%) 7 (4.1%) 9 (5.3%)	35 (43.8%) 13 (16.2%) 13 (16.2%) 19 (23.8%)
Grading^Δ^				
*G1* *G2* *G3* *p16-positive*	14 (5.9%) 166 (69.7%) 53 (22.3%) 5 (2.1%)	0 (0.0%) 4 (50%) 4 (50%) 0 (0.0%)	13 (7.8%) 130 (77.8%) 22 (13.2%) 2 (1.2%)	1 (1.3%) 40 (50.6%) 35 (44.3%) 3 (3.8%)
Lymphatic invasion^Δ^				
*L0* *L1*	210 (85.4%) 36 (14.6%)	8 (80%) 2 (20%)	157 (90.2%) 17 (9.8%)	61 (74.4%) 21 (25.6%)
Venous invasion^Δ^				
*V0* *V1*	218 (88.6%) 28 (11.4%)	9 (90%) 1 (10%)	161 (92.5%) 13 (7.5%)	66 (80.5%) 16 (19.5%)
Perineural invasion^Δ^				
*Pn0* *Pn1*	156 (65.3%) 83 (34.7%)	5 (50%) 5 (50%)	129 (76.8%) 39 (23.2%)	32 (39.5%) 49 (60.5%)
Follow-up [months]°	26.0 (11.2 – 45.8)	11.5 (6.2 – 27.0)	30.0 (17.0 – 48.8)	15.5 (6.0 – 35.2)
Local recurrence [events]^†^	37/246 (15.0%)	4/10 (40%)	20/174 (11.5%)	21/82 (25.6%)
Distant metastasis [events]^†^	13/246 (5.3%)	4/10 (40%)	6/174 (3.5%)	11/82 (13.4%)
Disease-specific death [events]^†^	14/244 (5.7%)	4/10 (40%)	6/174 (3.5%)	12/80 (15.0%)
Later lymph-node metastasis [events]^†^	13/246 (5.3%)	3/10 (30%)	5/174 (2.9%)	11/82 (13.4%)
Occult nodal metastasis among cN0 patients [events]^†^	27/174 (15.5%)	3/9 (33.3%)	10/131 (7.6%)	20/52 (38.5%)
Second HNSCC	36/246 (14.6%)	3/10 (30%)	26/174 (14.9%)	13/82 (15.9%)
Missing data^Δ^ *Age* *Gender* *Tumor localisation* *Tumor stage* *Nodal stage* *Grading* *Lymphatic invasion* *Venous invasion* *Perineural invasion* *Local recurrence* *Distant metastasis* *Disease-specific death* *Second HNSCC*	246 0 (0.0%) 0 (0.0%) 1 (0.4%) 0 (0.0%) 7 (2.8%) 8 (3.2%) 0 (0.0%) 0 (0.0%) 7 (2.8%) 0 (0.0%) 0 (0.0%) 2 (0.8%) 0 (0.0%)	10 0 (0.0%) 0 (0.0%) 0 (0.0%) 0 (0.0%) 0 (0.0%) 2 (20%) 0 (0.0%) 0 (0.0%) 0 (0.0%) 0 (0.0%) 0 (0.0%) 0 (0.0%) 0 (0.0%)	174 0 (0.0%) 0 (0.0%) 1 (0.6%) 0 (0.0%) 5 (2.9%) 7 (4.0%) 0 (0.0%) 0 (0.0%) 6 (3.5%) 0 (0.0%) 0 (0.0%) 0 (0.0%) 0 (0.0%)	82 0 (0.0%) 0 (0.0%) 0 (0.0%) 0 (0.0%) 2 (2.4%) 3 (3.6%) 0 (0.0%) 0 (0.0%) 1 (1.2%) 0 (0.0%) 0 (0.0%) 2 (2.4%) 0 (0.0%)

#Median (IQR) Δ Count (Frequency) °Median (IQR) †specific events/total events (Frequency).

### 2.2 Tissue sampling and histology preparation

Tissue samples and histochemical processing were performed as previously described by Leypold et al. (22). All patients were staged according to the 8^th^ edition of the TNM classification of the Union for International Cancer Control (UICC) (23), which was applied consistently across all included cases despite the subsequent release of a 9^th^ edition to ensure methodological uniformity (17). Histological processing was carried out at the Institute of Pathology, RWTH Aachen University, Germany, following surgical treatment at the Department of Oral and Maxillofacial Surgery. After cohort definition, formalin-fixed paraffin-embedded (FFPE) tissue blocks were sectioned at 4 µm thickness. Sections were stained with haematoxylin and eosin (HE) using eosin G solution (1% aqueous) and Mayer’s modified haematoxylin. Staining was performed using a Tissue-Tek Prisma automated staining system (Sakura, Umkirch, Germany).

### 2.3 Analysis of tumour budding in FFPE sections

Tumour budding (TB) was assessed on formalin-fixed, paraffin-embedded (FFPE) sections by two observers (JP and CC, board-certified pathologist specialized in head and neck pathology) in a consensus review. Both observers were blinded to clinical outcomes throughout the evaluation. Biopsy and resection specimens were assessed independently and observers were unaware of which biopsy corresponded to which resection specimen. All available slides for each case were initially screened to identify tumour-positive sections. The median number of evaluable tumour-containing slides was 1 per biopsy specimen (up to 5 slides) and 5 per resection specimen (up to 14 slides). For each section containing the tumour invasive front (TIF), a representative hotspot was selected at 10× magnification, and TB was scored at 20× magnification. One hotspot was evaluated per tumour-positive section. When serial sections from the same anatomical position were available, the highest TB score for that position was recorded. Sections containing previously decalcified bone tissue were included in the analysis, whereas slides with non-routine histochemical stains (e.g. PAS or EvG) were excluded. Tumour budding was defined as a single tumour cell or a cluster of up to four tumour cells, in accordance with the International Tumour Budding Consensus Conference criteria (6).

For each hotspot, TB was categorized into three groups according to the number of buds: score 1, 0–4 buds (low TB); score 2, 5–9 buds (intermediate TB); and score 3, ≥10 buds (high TB). Thus, the categorical thresholds were based on the ITBCC classification, while the present study extended the assessment by evaluating one hotspot per tumour-containing section rather than a single hotspot per specimen. A relative tumour budding score (TBrel) was calculated for each patient using the following formula:


$$TB_{rel}=\;\frac{\sum_{i=1}^{n}TB_{i}}{n}$$

where *TB_i_* is the categorical tumour-budding score for the *i*-th evaluable TIF section and *n* is the total number of analysed TIF section. Accordingly, *TBrel* represents the mean categorical TB score across evaluable tumour-containing sections, rather than an absolute number of tumour buds. This approach was adapted from the methodology proposed by Cacchi et al. (15, 24). Because resection specimens generally contained more evaluable tumour-containing sections than biopsy specimens, their assessment provided more sampling opportunities to identify areas with higher tumour budding, which represents a potential sampling-related consideration when comparing TBrel between specimen types. Digital slide images were acquired using a NanoZoomer S360MD slide scanner (Hamamatsu Photonics, Hamamatsu City, Japan).

### 2.4 Agreement between biopsy and resection tumour budding

Tumour budding was dichotomised in biopsy (preoperative incision biopsy) and resection specimens into low (≤1) and high (>1) groups (Biopsy TBlow n=246; Biopsy TBhigh n=10; Resection TBlow n=174; Resection TBhigh n=82). Agreement between biopsy and resection classifications was assessed using a contingency table. Overall agreement was calculated as the proportion of concordant classifications. Interobserver agreement beyond chance was quantified using Cohen’s kappa (κ) with 95% confidence intervals. In addition, sensitivity, specificity, positive predictive value (PPV), and negative predictive value (NPV) of biopsy-based classification were calculated using resection-based classification as reference standard.

### 2.5 Statistical analysis

All statistical analyses were performed using R version 4.3.3 (R Foundation for Statistical Computing, Vienna, Austria) within RStudio version 2024.04.2. Since biopsy and resection specimens were obtained from the same patients, all direct comparisons between specimen types were treated as paired observations. Continuous variables were assessed for distributional characteristics and are reported using descriptive summary statistics. For paired continuous TB measurements, the association between biopsy- and resection-derived measurements was evaluated using Spearman’s rank correlation coefficient, while within-patient differences were assessed using the Wilcoxon signed-rank test. For analyses based on dichotomized tumour budding categories, agreement between specimen types was examined using contingency tables, percentage agreement, Cohen’s kappa statistic and diagnostic performance metrics derived from confusion matrices. Differences in the frequency of dichotomized tumour budding between paired biopsy and resection specimens were assessed using McNemar’s test.

For analyses in which each patient contributed both a biopsy and a resection observation, associations between tumour budding and clinicopathological characteristics were evaluated using linear or logistic regression models, as appropriate, including specimen type and the corresponding specimen-type interaction term. To account for within-patient correlation between paired observations, standard errors were estimated using patient-clustered robust variance estimators, with patient ID as the clustering unit. For categorical variables with more than two levels, overall specimen-type interactions were assessed using joint Wald tests. Pairwise contrasts were adjusted for multiple comparisons where applicable.

To assess whether differences in sampling intensity contributed to the higher detection of tumour budding in resection specimens, an additional section-level sensitivity analysis was performed using the available PTTB scores. To approximate comparable section-level sampling between specimen types, one evaluable resection section was randomly selected for each patient and classified according to the same threshold. This procedure was repeated 5,000 times. For each iteration, the proportion of patients classified as high budding was calculated and compared with the observed biopsy classification. Results of the repeated sampling analysis are reported as the median and 2.5^th^–97.5^th^ percentiles across iterations; these percentile ranges reflect variation attributable to random section selection rather than conventional confidence intervals.

Time-to-event analyses were performed using the Kaplan-Meier method, with differences between groups assessed by the log-rank test. Disease-specific survival was defined as the interval from surgery to disease-specific death or last follow-up. Patients without an event were censored at the date of last follow-up. For local recurrence, distant metastasis and later lymph-node metastasis, time to event was calculated from primary treatment to the respective event. Patients who did not experience the endpoint were censored at their individual last documented follow-up. Thus, event-free patients were assigned their observed follow-up duration rather than an endpoint time of zero. The prognostic value of tumour budding classification was evaluated using Cox proportional hazards regression. Separate univariable models were fitted for biopsy- and resection-derived tumour budding. A combined multivariable model was subsequently constructed including both biopsy- and resection-derived tumour budding simultaneously as patient-level predictors. Effect estimates are reported as hazard ratios (HRs) with 95% confidence intervals (CIs). The incremental prognostic contribution of each specimen type was evaluated by likelihood ratio testing of nested models. Model discrimination was assessed using Harrell’s concordance index (C-index). Associations between categorical variables were evaluated using Pearson’s chi-squared test, with Fisher’s exact test applied when expected cell counts were insufficient. To investigate the relationship between tumour budding and binary clinical outcomes, logistic regression models were fitted. Univariable models were constructed for each specimen type separately, followed by a combined model incorporating both assessments. The combined logistic regression model incorporated both specimen-derived measurements at the patient level. Results are presented as odds ratios (ORs) with 95% confidence intervals. Incremental predictive value was assessed by comparing nested logistic regression models using likelihood ratio tests. All statistical tests were two-sided and p-values < 0.05 were considered statistically significant. Missing data were handled by complete-case analysis for each individual statistical procedure.

### 2.6 Ethical statement

This study received approval from the local ethics committee (Ethics Committee of the University Hospital RWTH Aachen, approval number EK 22-373) and was conducted in accordance with Good Clinical Practice guidelines and the Declaration of Helsinki.

## 3 Results

### 3.1 Biopsy assessment underestimates tumour budding

A total of 256 patients with available paired biopsy and resection specimens were included in the analysis (**Figure 1**, **Table 1**). All available tumour-containing sections from biopsy and resection specimens were assessed, with one representative tumour-budding hotspot evaluated per section as previously established (15, 22). Biopsy- and resection-derived TBrel scores showed a weak positive association (Spearman ρ = 0.235, p < 0.001, n = 256 paired observations; **Figures 2A, B**). Tumour budding classification showed moderate overall concordance between biopsy and resection specimens, with an overall agreement of 70.3% (180/256 cases). However, agreement beyond chance was low (Cohen’s κ = 0.11, 95% CI 0.03–0.20). Cross-tabulation demonstrated a strong imbalance in misclassification patterns. While most low-budding tumours were consistently classified as low in both biopsy and resection (172 cases), high tumour budding was frequently underdetected in biopsy specimens. Of 82 cases classified as high budding in resection specimens, only 8 were correctly identified in biopsy, corresponding to a sensitivity of 9.8%. Specificity was high (98.9%), reflecting a strong tendency of biopsy to classify cases as low budding. McNemar’s test indicated significant asymmetry in misclassification (p = 3.8×10^–16^), consistent with systematic underdetection of high tumour budding in biopsy specimens.

**Figure 1:**
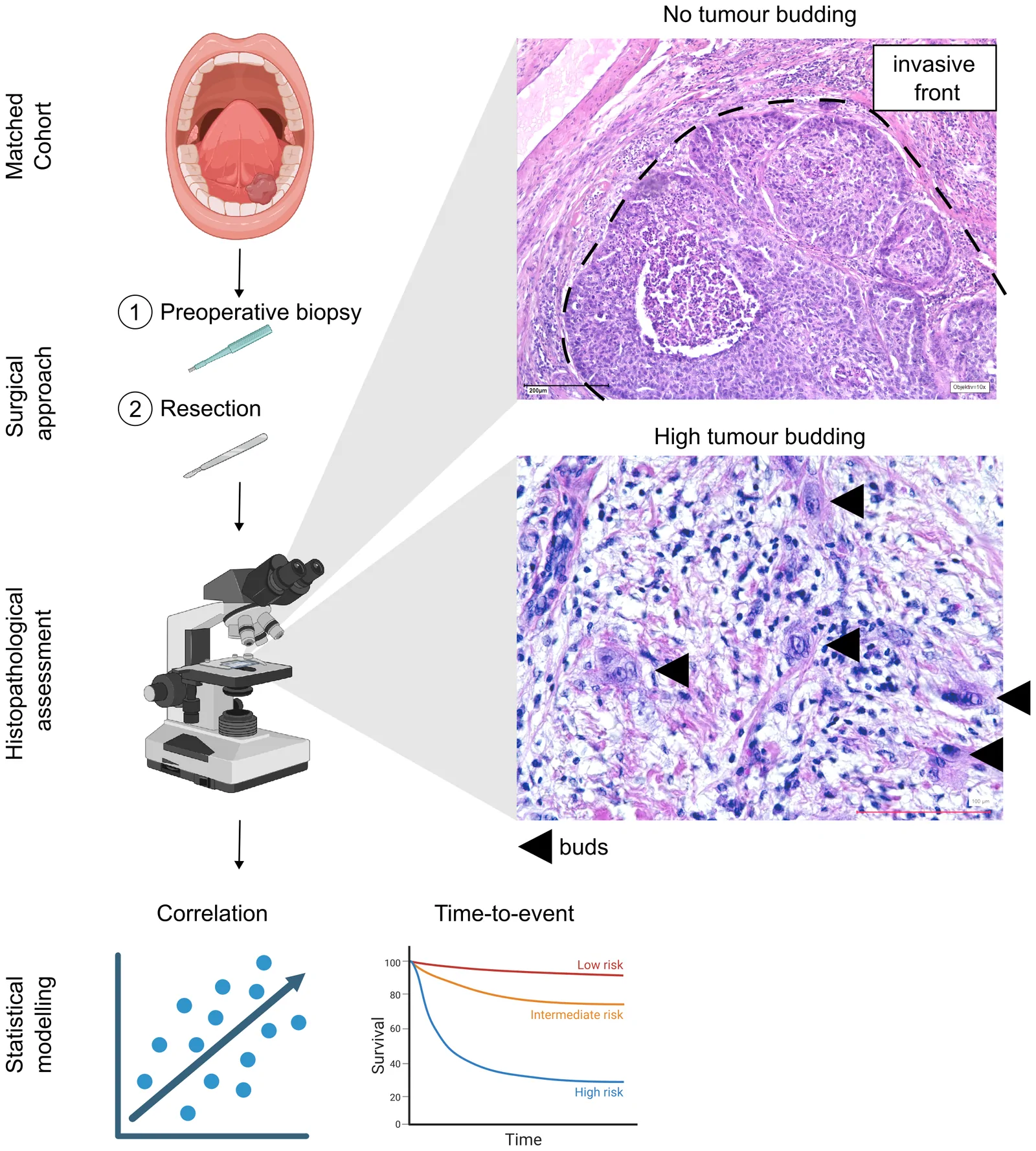
Visual abstract. Patients with oral squamous cell carcinoma (OSCC) underwent a preoperative incisional biopsy followed by surgical resection. Only cases with matched biopsy and resection specimens were included. Tumour budding was assessed at the invasive front on all available slides from both specimen types, and relative tumour budding (TBrel) was calculated. Representative H&E-stained images are shown; the dotted line indicates the invasive front, and arrowheads highlight selected tumour buds in a case with high tumour budding. The black scale bar represents 200 µm. Statistical analyses were performed to evaluate the correlation between tumour budding in biopsy and resection specimens and its prognostic value for survival outcomes.

**Figure 2:**
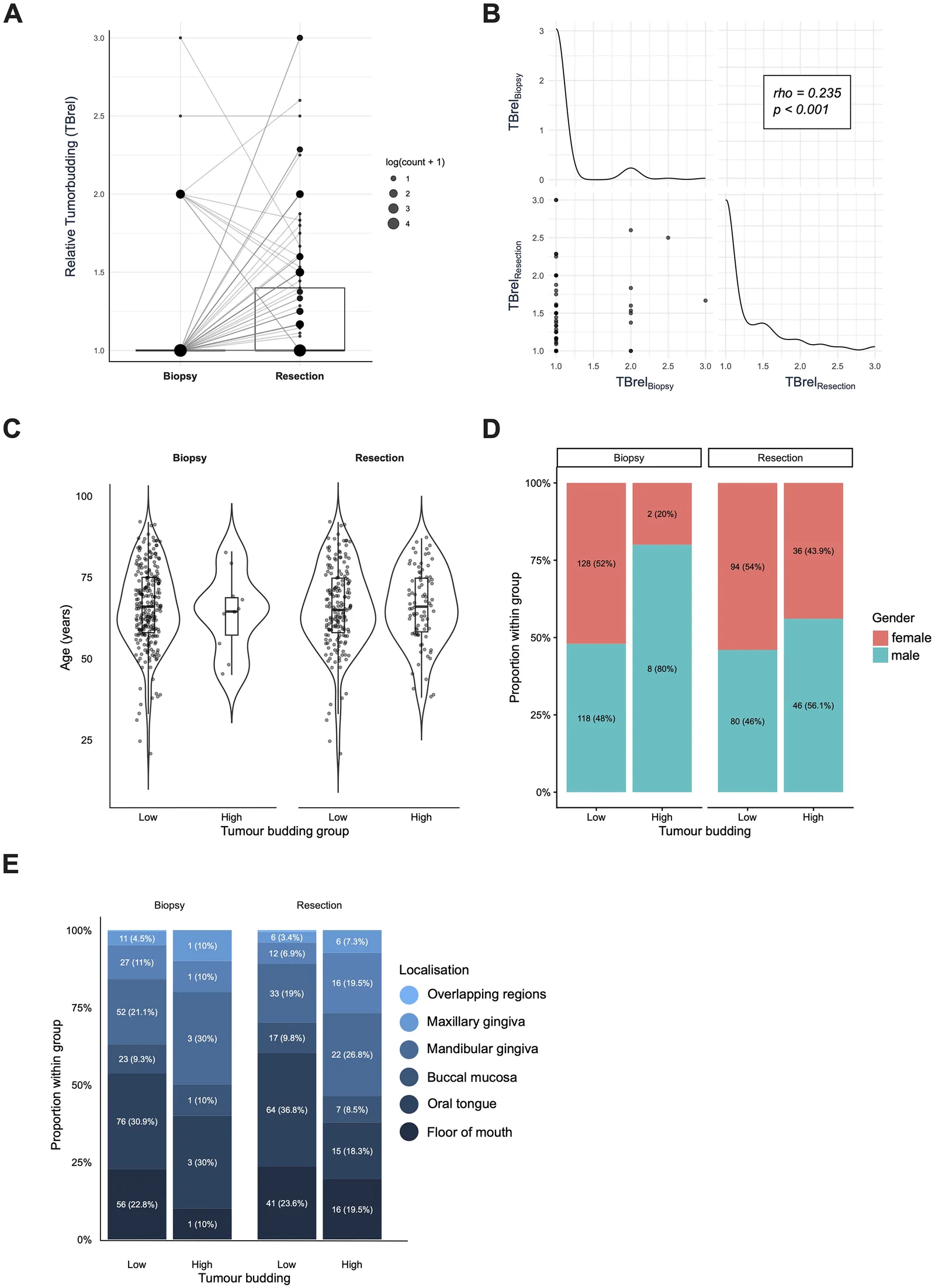
Agreement between tumour budding in biopsy and resection specimens and patient characteristics. **(A)** Boxplot with paired observations showing tumour budding **(TBrel)** in matched biopsy and resection specimens. Each line connects matched specimens. Point size is scaled according to log(count + 1). **(B)** Histograms and scatterplot illustrating the relationship between TBrel in matched biopsy and resection specimens. Spearman’s correlation coefficient is shown. **(C)** Violin plots with overlaid boxplots and individual patient observations showing age according to low- and high-TBrel groups. Each patient is represented twice (biopsy and matched resection). **(D)** Stacked bar plot showing sex distribution across TBrel-defined groups. Counts are indicated within each bar. **(E)** Stacked bar plot showing tumour localization across TBrel-defined groups. Regression analyses for panels C–E accounted for paired biopsy and resection observations using patient-clustered robust standard errors.

To assess the contribution of unequal sampling intensity, we performed a section-level sensitivity analysis restricted to the 247 patients with at least two evaluable resection sections. Nine patients with only one evaluable resection section were excluded; this exclusion was based solely on section availability, although all nine were classified as low budding in the primary resection analysis. Among the 247 included patients, 10 biopsies (4.0%) were classified as high budding. In each of 5,000 iterations, one evaluable resection section was selected at random for each patient. The median proportion classified as high budding was 15.8%, with a 2.5th–97.5th percentile simulation range of 12.6%–19.0%. When all evaluated resection sections were considered in this subgroup, 82 of 247 patients (33.2%) were classified as high budding; the corresponding frequency in the full primary cohort was 82 of 256 (32.0%). Thus, limiting resection assessment to a single section substantially reduced the detection of high budding, suggesting that sampling intensity contributed to the difference between biopsy- and resection-based classifications. Nevertheless, high budding remained more frequently detected in a randomly selected resection section than in the corresponding biopsy assessment. This residual difference suggests that section number alone did not fully explain the discordance, although the analysis cannot distinguish the effects of sampled tissue area, anatomical targeting, and biological heterogeneity.

### 3.2 Associations between tumour budding and clinicopathological characteristics

To assess whether associations between tumour budding and clinicopathological characteristics differed between biopsy and resection specimens, regression models including specimen-type interaction terms were fitted with patient-clustered robust standard errors to account for the paired observations contributed by each patient. In the analysis of age, there was no evidence that the association between tumour budding and age differed between biopsy and resection specimens (interaction coefficient 2.20 years, cluster-robust SE 3.65, p = 0.547; **Figure 2C**). Similarly, no evidence of a specimen-dependent association with gender was observed (interaction OR = 0.35, 95% CI 0.07–1.65, p = 0.183; **Figure 2D**). Tumour location was not associated with biopsy-derived high tumour budding in any of the multiplicity-adjusted pairwise comparisons. In resection specimens, maxillary-gingival tumours had higher odds of high tumour budding than oral-tongue tumours (OR = 5.69, 95% CI 1.37–23.54, multiplicity-adjusted p = 0.005), whereas no other pairwise comparison was statistically significant. However, the joint tumour-location-by-specimen interaction was not significant (Wald χ² = 2.80, 5 df, p = 0.732), providing no evidence that the overall association between tumour location and tumour budding differed between specimen types (**Figure 2E**). These estimates should be interpreted cautiously because of the small number of high-budding biopsy specimens and limited numbers in several anatomical subgroups.

For pT stage, high tumour budding in resection specimens was more frequent in pT2–pT4 tumours (42.9%–49.2%) than in pT1 tumours (6.7%). Compared with pT1, the odds of high resection-derived tumour budding were higher for pT2 (OR = 11.03, 95% CI 2.94–41.45), pT3 (OR = 10.50, 95% CI 2.59–42.49), and pT4 tumours (OR = 13.58, 95% CI 3.68–50.03; all multiplicity-adjusted p < 0.001). No corresponding pairwise comparison was statistically significant for biopsy-derived tumour budding, although these estimates were imprecise because only 10 biopsies were classified as high budding. The joint pT-stage-by-specimen interaction was not statistically significant (Wald χ² = 1.40, 3 df, p = 0.706), providing no evidence that the overall association between pT stage and tumour budding differed between biopsy and resection specimens (**Figure 3A**).

**Figure 3:**
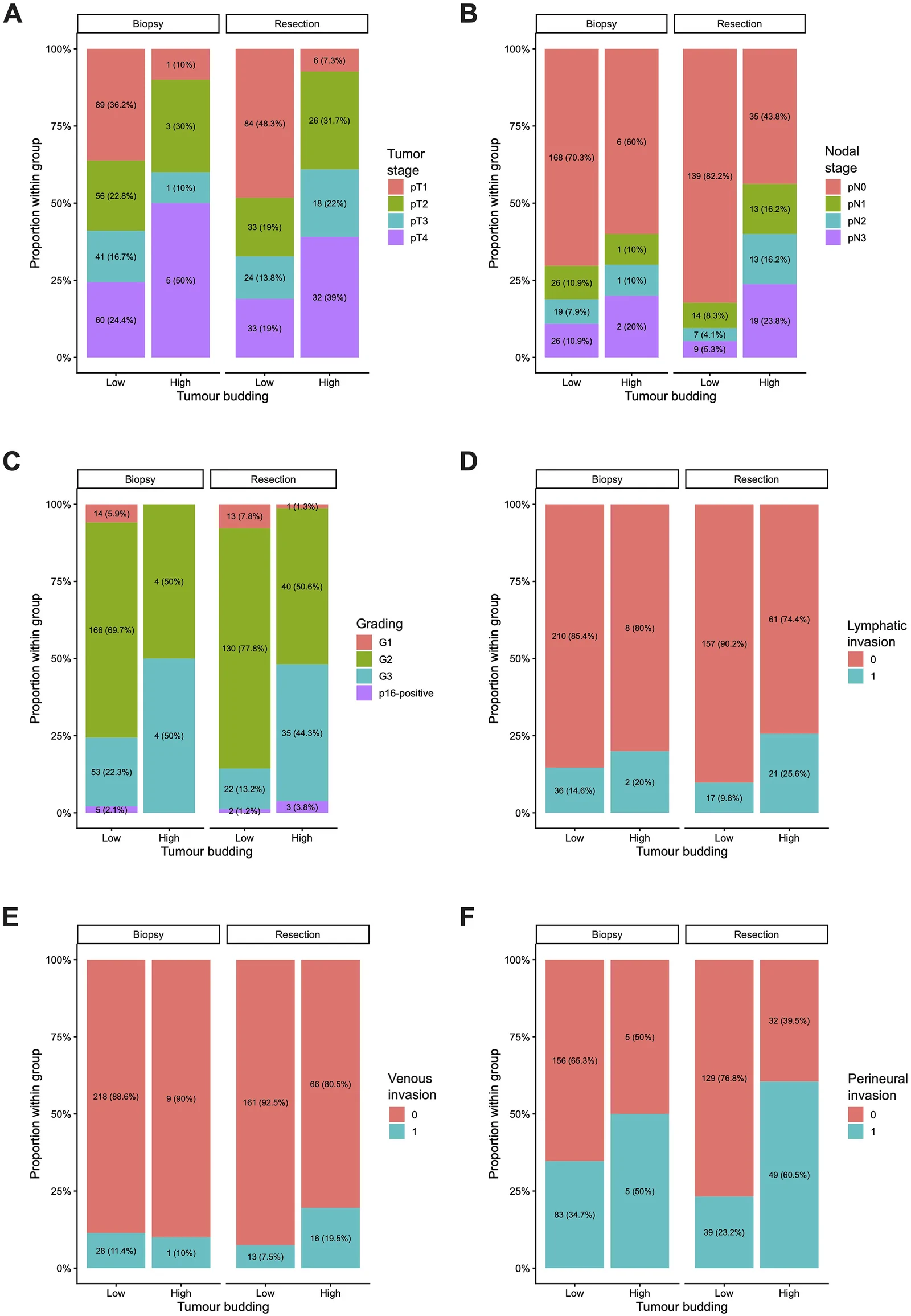
Clinicopathological characteristics according to tumour budding classification. **(A)** Distribution of pathological T stage across TBrel-defined groups in matched biopsy and resection specimens. **(B)** Distribution of pathological N stage across TBrel-defined groups. **(C)** Distribution of tumour grade across TBrel-defined groups. Histological grading was not performed for p16-positive tumours, no regression was calculated. **(D)** Distribution of lymphatic invasion across TBrel-defined groups. **(E)** Distribution of venous invasion across TBrel-defined groups. **(F)** Distribution of perineural invasion across TBrel-defined groups. Regression analyses comparing associations across specimen types accounted for the paired biopsy and resection observations using patient-clustered robust standard errors.

For pN-stage data, high tumour budding in resection specimens increased from 20.1% in pN0 tumours to 48.1% in pN1, 65.0% in pN2 and 67.9% in pN3 tumours. Compared with pN0, the odds of high resection-derived tumour budding were higher for pN1 (OR = 3.69, 95% CI 1.18–11.56, multiplicity-adjusted p = 0.010), pN2 (OR = 7.38, 95% CI 1.92–28.33, p < 0.001), and pN3 tumours (OR = 8.38, 95% CI 2.55–27.54, p < 0.001). No corresponding pairwise comparison was statistically significant for biopsy-derived tumour budding, again with limited precision because of the small number of high-budding biopsies. The joint pN-stage-by-specimen interaction was not statistically significant (Wald χ² = 4.23, 3 df, p = 0.237), providing no evidence that the overall association between pN stage and tumour budding differed between specimen types (**Figure 3B**).

The absence of high tumour budding in the G1 and p16-positive biopsy groups resulted in separation, rendering the conventional logistic interaction model unreliable. Accordingly, grading was reported descriptively and the available data do not permit reliable inference regarding whether the association between grading and tumour budding differs between specimen types.

Lymphatic invasion was not significantly associated with high tumour budding in biopsy specimens (OR = 1.46, 95% CI 0.30–7.20, p = 0.643), whereas high resection-derived tumour budding was more frequent in tumours with lymphatic invasion (55.3% vs. 28.0%; OR = 3.18, 95% CI 1.57–6.45, p = 0.001). The lymphatic-invasion-by-specimen interaction was not statistically significant (interaction OR = 2.18, 95% CI 0.44–10.85, p = 0.341).

Similarly, venous invasion was not significantly associated with high tumour budding in biopsy specimens (OR = 0.87, 95% CI 0.10–7.16, p = 0.893), whereas in resection specimens high tumour budding was more frequent in tumours with venous invasion than in those without venous invasion (55.2% vs. 29.1%; OR = 3.00, 95% CI 1.36–6.61, p = 0.006). The venous-invasion-by-specimen interaction was not statistically significant (interaction OR = 3.47, 95% CI 0.42–28.98, p = 0.250).

Perineural invasion was likewise not significantly associated with high tumour budding in biopsy specimens (OR = 1.88, 95% CI 0.53–6.72, p = 0.332), whereas high resection-derived tumour budding was substantially more frequent in tumours with perineural invasion than in those without it (55.7% vs. 19.9%; OR = 5.06, 95% CI 2.85–9.00, p < 0.001). The perineural-invasion-by-specimen interaction was not statistically significant (interaction OR = 2.69, 95% CI 0.74–9.83, p = 0.133) (**Figures 3C–F**).

For venous invasion, lymphatic invasion, and perineural invasion, the interaction confidence intervals were wide. Thus, the absence of statistically significant interaction should not be interpreted as evidence that specimen-dependent differences are absent, and clinically relevant differences cannot be excluded.

### 3.3 Prognostic value of biopsy-derived tumour budding for risk stratification

Despite the systematic underestimation of tumour budding in biopsy compared with resection specimens, we assessed whether biopsy-derived tumour budding retains prognostic value. Both biopsy (log-rank test, p<0.001) and resection (log-rank test, p<0.001) tumour budding were strong predictors of disease-specific survival (**Figures 4A, B**). In the biopsy cohort, the high-budding group comprised 10 patients, of whom 4 experienced a disease-specific event (40%), compared with 14 events among the 244 patients (5.7%) in the low-budding group. In the resection cohort, the high-budding group comprised 80 patients with 12 disease-specific events (15%), compared with 6 events among 174 patients (3.4%) in the low-budding group. High biopsy-derived tumour budding was also associated with an increased probability of subsequent distant metastasis (log-rank test, p<0.0001; **Figure 4C**) and local recurrence (log-rank test, p=0.0075; **Figure 4D**). Given the small number of patients in the biopsy high-budding group and the limited number of events, these findings should be interpreted cautiously.

**Figure 4:**
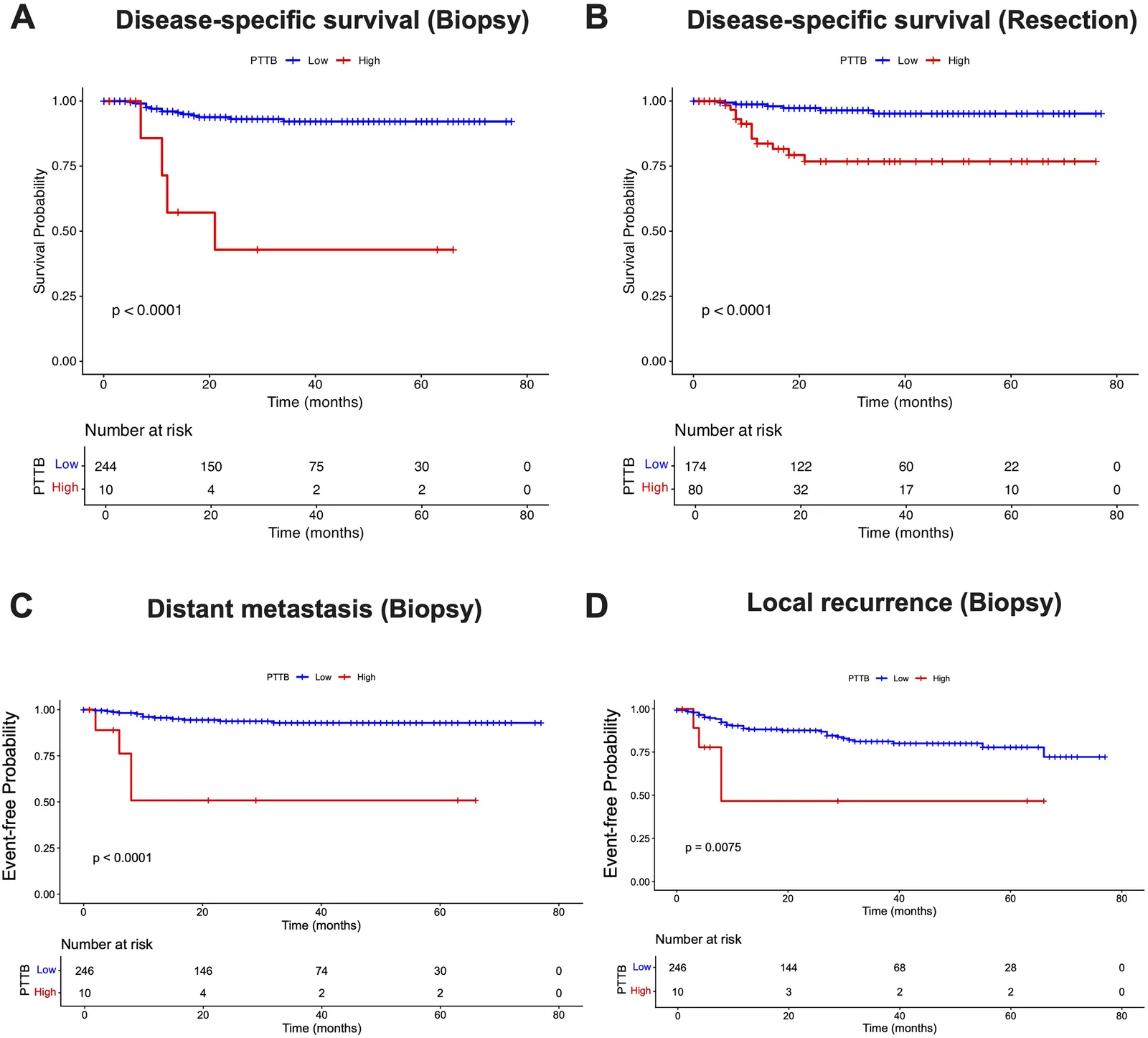
Prognostic impact of tumour budding in biopsy and resection specimens. **(A)** Kaplan–Meier analysis of disease-specific survival (DSS) according to biopsy-derived TBrel. **(B)** Kaplan–Meier analysis of DSS according to resection-derived TBrel. **(C)** Kaplan–Meier analysis of distant metastasis-free probability after primary resection according to biopsy-derived TBrel. **(D)** Kaplan–Meier analysis of local recurrence-free probability according to biopsy-derived TBrel. P-values were calculated using the log-rank test. Time was measured from the date of surgical resection (months).

### 3.4 Uni- and multivariable analyses of tumour budding for survival and metastasis outcomes

In univariable analysis, low tumour budding in biopsy specimens was associated with a substantial reduction in disease-specific mortality risk compared with high budding (HR 0.10, 95% CI 0.03–0.31, p < 0.001; **Figure 5A**). The corresponding model had a Harrell’s C-index of 0.60 (SE 0.05). Similarly, low tumour budding in resection specimens was associated with improved disease-specific survival (HR 0.16, 95% CI 0.06–0.43, p < 0.001), with a C-index of 0.72 (SE 0.05). Although the point estimate for discrimination was higher for the resection-based model, no formal comparison of the C-indices was performed. Given the small number of patients and events in the biopsy high-budding group, the corresponding hazard ratio should be interpreted with caution, as the limited sample size may result in reduced precision of the estimate. In a multivariable Cox model including both biopsy and resection tumour budding, both variables remained associated with disease-specific survival after mutual adjustment. Low tumour budding in biopsy specimens remained significantly associated with improved DSS (HR = 0.17, 95%-CI 0.05–0.54, p = 0.003), as did low tumour budding in resection specimens (HR = 0.20, 95%-CI 0.07–0.54, p = 0.002). The combined model had a C-index of 0.74 (SE 0.06), compared with 0.60 (SE 0.05) for biopsy tumour budding alone and 0.72 (SE 0.05) for resection tumour budding alone. Likelihood ratio testing demonstrated that each specimen type provided additional prognostic information conditional on the other. Adding resection tumour budding to a model containing biopsy tumour budding significantly improved model fit (χ² = 10.55, p = 0.0012), while adding biopsy tumour budding to a model containing resection tumour budding also improved model fit (χ² = 6.54, p = 0.011). However, because the increase in the apparent C-index was small when biopsy tumour budding was added to the resection-based model and because the number of disease-specific events was limited, these findings should be interpreted as evidence of incremental model fit rather than definitive improvement in prognostic discrimination.

**Figure 5:**
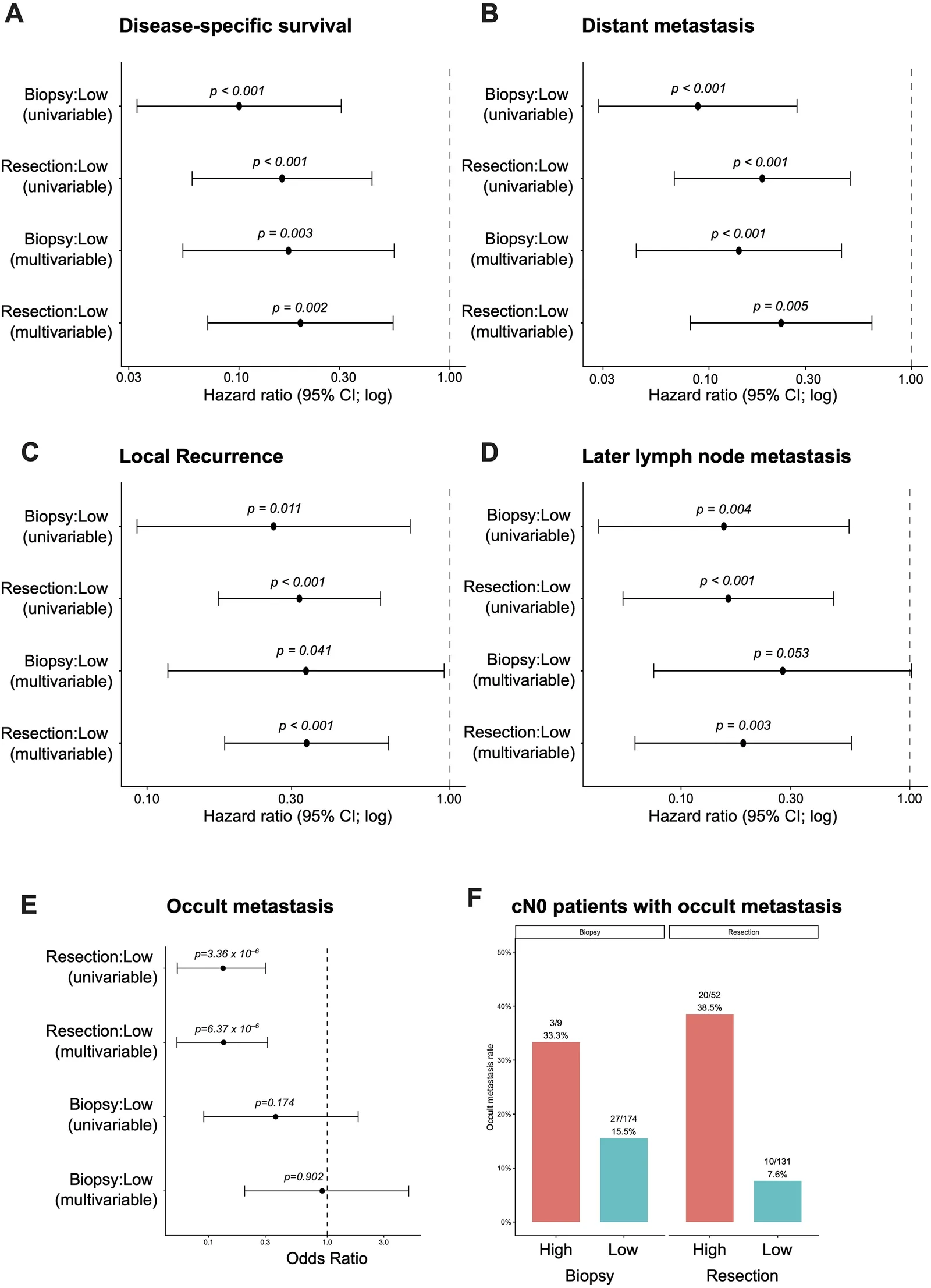
Univariable and multivariable analyses of tumour budding. **(A)** Forest plot showing the association of tumour budding classification (low vs. high) with disease-specific survival **(DSS)** in univariable and multivariable Cox regression models. **(B)** Forest plot showing the association with distant metastasis developing after primary resection. **(C)** Forest plot showing the association with local recurrence. **(D)** Forest plot showing the association with lymph node metastasis developing after primary resection. **(E)** Forest plot showing the association with occult nodal metastasis (cN0/pN+) in logistic regression models. High tumour budding served as the reference category in all regression analyses. **(F)** Proportion of patients with occult nodal metastasis across TBrel-defined groups. Numbers above the bars indicate patients with occult metastasis/total number of patients in each group and frequency.

Distant metastasis occurred in 4 of 10 patients in the high-budding biopsy group (40%) and in 13 of 246 patients in the low-budding biopsy group (5%). Low biopsy-derived tumour budding was associated with a markedly lower hazard of distant metastasis in univariable analysis (HR = 0.09, 95% CI 0.03–0.27, p<0.001; **Figure 5B**). Low resection-derived tumour budding was likewise associated with a lower hazard of distant metastasis (HR = 0.18, 95% CI 0.07–0.50, p<0.001). After mutual adjustment, both biopsy-derived tumour budding (HR = 0.14, 95% CI 0.04–0.45, p<0.001) and resection-derived tumour budding (HR = 0.23, 95% CI 0.08–0.64, p=0.0049) remained significantly associated with distant metastasis. The combined model had a C-index of 0.73. Adding resection tumour budding to the biopsy-based model significantly improved model fit (χ²=8.25, p=0.0041) and adding biopsy tumour budding to the resection-based model also improved model fit (χ²=7.74, p=0.0054). These likelihood-ratio tests indicate incremental prognostic information conditional on the other specimen type and should not be interpreted as demonstrating superior discrimination of either model.

Local recurrence occurred in 4 of 10 patients (40%) in the high-budding biopsy group and in 37 of 246 patients (15%) in the low-budding biopsy group. Low tumour budding was associated with a lower hazard in both the biopsy-based univariable model (HR = 0.26, 95% CI 0.09–0.74, p=0.011; **Figure 5C**) and the resection-based univariable model (HR = 0.32, 95% CI 0.17–0.59, p<0.001). At the coefficient level, both associations remained statistically significant by Wald testing after mutual adjustment (biopsy: HR = 0.33, 95% CI 0.12–0.96, p=0.041; resection: HR = 0.34, 95% CI 0.18–0.63, p<0.001). The combined model had a C-index of 0.65. Adding resection tumour budding to the biopsy-based model significantly improved model fit (χ²=11.29, p<0.001), whereas adding biopsy tumour budding to the resection-based model did not result in a statistically significant improvement (χ²=3.18, p=0.074). Thus, for local recurrence, resection tumour budding provided additional prognostic information beyond biopsy tumour budding, whereas there was insufficient evidence that biopsy tumour budding added information beyond the resection-based model.

For later lymph-node metastasis, low tumour budding was associated with a reduced hazard in both biopsy specimens (HR = 0.15, 95% CI 0.04–0.54, p=0.0036; **Figure 5D**) and resection specimens (HR = 0.16, 95% CI 0.06–0.46, p<0.001) in univariable analyses. After mutual adjustment, resection-derived tumour budding remained significantly associated with later lymph-node metastasis (HR = 0.19, 95% CI 0.06–0.56, p=0.0025), whereas the association with biopsy-derived tumour budding was attenuated and did not reach conventional statistical significance (HR = 0.28, 95% CI 0.08–1.02, p=0.053). The combined model had a C-index of 0.73. Adding resection tumour budding to the biopsy-based model significantly improved model fit (χ²=9.82, p=0.0017), whereas adding biopsy tumour budding to the resection-based model did not (χ²=2.92, p=0.088). This pattern indicates stronger evidence for incremental prognostic information from resection tumour budding in this model, without establishing superior discriminative performance.

Although these analyses demonstrate consistent associations between tumour budding and secondary oncological outcomes, the distant-metastasis and later lymph-node-metastasis models were based on a limited number of events, particularly within the high-budding biopsy subgroup. Estimates from these analyses should therefore be interpreted cautiously because of their limited precision.

### 3.5 Occult metastasis

Occult nodal metastasis, defined as histologically confirmed lymph node metastasis in patients without clinical evidence of nodal disease (cN0). The occult nodal metastasis analysis included 183 cN0 patients with available pathological nodal status following neck dissection. Of the 10 patients classified as high tumour budding based on biopsy specimens, 9 were cN0 and underwent neck dissection and were therefore included in the occult metastasis analysis; the remaining patient was clinically node-positive (cN+) and was excluded. In clinically node-negative patients, low resection-derived tumour budding was associated with lower odds of occult nodal metastasis relative to high budding (OR 0.19, p < 0.001; **Figure 5E**), whereas biopsy-derived tumour budding showed only a non-significant trend (p = 0.174). In multivariable analysis, only resection-based tumour budding remained associated with occult nodal disease after mutual adjustment and significantly improved model fit (p < 0.001), while adding biopsy-derived tumour budding did not significantly improve model fit. Risk stratification further highlighted this difference: biopsy-based classification identified nine patients as high risk, of whom three patients (33.3%) had occult metastases, while 27 of 174 patients (15.5%) classified as low risk also harboured occult disease. In contrast, resection-based classification showed clearer discrimination, with 20 of 52 high-risk patients (38.5%) exhibiting occult metastasis compared with 10 of 131 low-risk patients (7.6%) (**Figure 5F**).

## 4 Discussion

In the present study, we evaluated the concordance and comparative prognostic value of tumour budding assessed in preoperative biopsies and matched resection specimens in a cohort of 256 patients. We demonstrate four principal findings. First, categorical agreement between biopsy and resection tumour budding was poor and driven by systematic underdetection of high-grade budding in biopsies. Second, both biopsy- and resection-derived tumour budding were associated with disease-specific survival, with the resection-based assessment showing a numerically higher C-index than the biopsy-based assessment. Third, biopsy- and resection-derived tumour budding were associated with distant metastasis and local recurrence after mutual adjustment. Fourth, resection-derived tumour budding remained significantly associated with later lymph-node metastasis after mutual adjustment, whereas the association with biopsy-derived tumour budding was attenuated, although secondary endpoint analyses remain exploratory. Despite numerical differences in C-index estimates, biopsy and resection tumour budding provided partially overlapping prognostic information when included simultaneously in the same model. This issue is particularly timely in light of the 9^th^ edition of the UICC TNM Classification of Malignant Tumours, which acknowledges tumour budding as an additional tumour-related prognostic factor but does not define a standardized assessment strategy (17). Our findings indicate that specimen type represents a critical methodological variable: biopsy-derived tumour budding may identify a subset of biologically aggressive tumours preoperatively, but its low sensitivity precludes its use as a surrogate for resection-based assessment.

Tumour budding is increasingly recognized as one of the most robust histopathological prognostic markers in oral squamous cell carcinoma (OSCC) (16). Multiple systematic reviews and meta-analyses have consistently demonstrated associations between high tumour budding, lymph node metastasis, recurrence and reduced survival, supporting its incorporation into routine pathological risk assessment (12–14). Nevertheless, most available evidence is based on resection specimens, whereas the clinical need is to identify high-risk patients before definitive surgery. Consequently, the utility of tumour budding assessment in preoperative biopsies remains of high clinical interest.

Only a limited number of studies have investigated biopsy-derived tumour budding. In colorectal adenocarcinoma, biopsy tumour budding has repeatedly shown prognostic value and reasonable concordance with resection findings, although sampling variability remains a recognized limitation (18, 19). Similar observations have recently been reported in pulmonary squamous cell carcinoma, where biopsy-derived tumour budding retained prognostic significance but incompletely represented the tumour budding status of the entire resection specimen (20). In OSCC, Xie et al. demonstrated that tumour budding assessed in pretreatment biopsies predicts cervical lymph node metastasis and survival, supporting its potential value for preoperative risk stratification (24). Our study substantially extends these observations by directly comparing matched biopsy and resection specimens in a large cohort while evaluating both morphological concordance and prognostic performance.

A key observation of our study is the marked discordance between biopsy and resection assessment. Although overall agreement appeared moderate, this was largely driven by correct classification of low-budding tumours, whereas biopsy failed to identify the majority of high-budding cases, resulting in a sensitivity of only approximately 10%. Our section-level sensitivity analysis demonstrates that unequal sampling intensity explains an important part of this discrepancy. Examining a larger number of resection sections substantially increases the probability of detecting a focal high-budding area. This sampling advantage is inherent to resection-based assessment which permits examination of a much broader extent of the invasive tumour front, whereas an incisional biopsy necessarily captures only a limited portion of the tumour–host interface. Biopsies may also incompletely represent the deepest invasive front, a region of particular relevance for tumour budding assessment and a potential site of focally increased budding. Accordingly, the residual difference observed even after restricting resection assessment to a single section should not be attributed exclusively to biological heterogeneity. It may additionally reflect anatomical sampling, tissue volume, the probability of including the deepest invasive front, and the spatial distribution of budding within the tumour. Previous reviews have highlighted tumour heterogeneity and sampling bias as important challenges for biopsy-based assessment (25) and our findings provide quantitative evidence supporting these concerns. The very low Cohen’s κ further demonstrates that apparently acceptable raw agreement substantially overestimates true concordance between biopsy and resection specimens.

Despite this morphological discordance, both biopsy- and resection-derived tumour budding showed statistically significant associations with disease-specific survival. These findings agree with previous meta-analyses demonstrating that tumour budding is among the strongest histological predictors of outcome in OSCC regardless of differences in scoring methodology (13, 14).

The C-index point estimate was numerically higher for resection-derived tumour budding than for biopsy-derived tumour budding (0.72 vs. 0.60). However, despite reporting the standard errors of these estimates, no formal paired statistical comparison of the C-indices was performed; the numerical difference should therefore not be interpreted as evidence of superior discrimination. Resection specimens permit more comprehensive assessment of the invasive front and may therefore capture tumour budding heterogeneity more completely than limited biopsy specimens. This observation also supports recent efforts advocating whole-invasive-front assessment rather than hotspot-based evaluation whenever technically feasible.

Importantly, multivariable modelling demonstrated that biopsy and resection tumour budding were not fully redundant. Both remained associated with disease-specific survival after mutual adjustment and each significantly improved model fit beyond the other. However, the apparent increase in C-index was modest, indicating that biopsy and resection capture largely overlapping biological information while retaining a limited amount of prognostic signal after mutual adjustment. These findings suggest that biopsy-derived tumour budding reflects the underlying invasive phenotype despite incomplete sampling of the invasive front.

The time-to-event analyses demonstrated that the prognostic relevance of tumour budding was not restricted to disease-specific survival. Both biopsy- and resection-derived tumour budding were associated with distant metastasis and local recurrence in univariable and mutually adjusted Cox models. For distant metastasis, each specimen type also significantly improved model fit beyond the other, suggesting partially complementary prognostic information. For local recurrence, however, adding resection-derived tumour budding significantly improved the biopsy-based model, whereas adding biopsy-derived tumour budding did not significantly improve the resection-based model. These results suggest that resection assessment may capture local-recurrence risk more comprehensively, although biopsy assessment retained an association with local recurrence after mutual adjustment. A related pattern was observed for later lymph-node metastasis. Both biopsy- and resection-derived tumour budding were significant in univariable analyses, but only resection-derived tumour budding remained significant after mutual adjustment. The biopsy-derived estimate was attenuated and narrowly crossed the null value. Furthermore, adding resection tumour budding significantly improved the biopsy-based model, whereas adding biopsy tumour budding did not significantly improve the resection-based model. These findings are consistent with the more extensive sampling of the invasive front afforded by resection specimens. However, because only 16 later lymph-node metastases and 17 distant metastases occurred, these secondary analyses remain susceptible to imprecision and model instability and should be interpreted as exploratory.

The relationship between tumour budding and occult cervical lymph node metastasis deserves particular consideration. Previous studies and recent meta-analyses have consistently associated high tumour budding with nodal metastasis in OSCC (14). In our analysis of occult nodal disease at primary treatment, only resection-derived tumour budding predicted occult nodal metastasis after mutual adjustment, whereas biopsy-derived tumour budding showed only a non-significant trend. A similar specimen-dependent pattern was observed for later lymph-node metastasis: although both biopsy- and resection-derived tumour budding were associated with the endpoint in univariable analyses, only resection-derived tumour budding remained significant after mutual adjustment. This discrepancy is likely explained by the systematic underestimation of high-grade budding in biopsy specimens rather than by a fundamentally different biological association. Because nodal dissemination may be driven by the most invasive tumour regions, incomplete sampling of the invasive front may disproportionately reduce the predictive performance of biopsy-derived assessment for this endpoint.

From a clinical perspective, these findings are particularly relevant for preoperative decision-making. The clinically important question is not whether biopsy and resection tumour budding are morphologically identical, but whether biopsy-derived assessment provides sufficient prognostic information before surgery. Our results indicate that despite substantial underdetection of high tumour budding, biopsy assessment retains clinically meaningful prognostic information and achieves significant risk stratification. This finding is consistent with previous biopsy-based studies in OSCC and other tumour entities and supports the concept that the biological processes reflected by tumour budding are already detectable in limited pretreatment tissue samples.

However, biopsy-derived tumour budding should not be regarded as a direct surrogate for resection-based assessment. The very low sensitivity for high tumour budding indicates that a clinically relevant subset of aggressive tumours will be misclassified if biopsy findings are interpreted in isolation. This limitation may be particularly important when tumour budding is used to guide treatment escalation, selection of patients for elective neck dissection or neoadjuvant therapy or inclusion into risk-adapted clinical trials. Consequently, biopsy tumour budding should currently be considered a pragmatic preoperative biomarker rather than a replacement for comprehensive postoperative assessment.

Several limitations should be acknowledged. This was a retrospective single-centre study and external validation in independent multicentre cohorts is warranted. Furthermore, although observers were blinded and assessment followed International Tumour Budding Consensus Conference criteria, tumour budding scoring remains partly observer dependent, emphasizing the need for continued standardization and potentially digital image analysis approaches. Although high intra- and inter-observer agreements in tumour budding evaluation have been repeatedly shown, observer-related variability remains an inherent limitation of histopathological assessment (24, 26). Median follow-up differed between tumour budding groups. In biopsy specimens, median follow-up was 26.0 months (IQR 11.2–45.8) in the low-budding group and 11.5 months (IQR 6.2–27.0) in the high-budding group. In resection specimens, corresponding values were 30.0 months (IQR 17.0–48.8) and 15.5 months (IQR 6.0–35.2), respectively. The shorter observed follow-up in the high-budding groups should be considered when interpreting outcome analyses; however, it may also partly reflect earlier occurrence of disease-related events rather than differences in surveillance alone. The relatively small number of patients classified as high tumour budding on biopsy (n = 10) also limits the precision and stability of estimates involving this subgroup, particularly for survival and secondary outcome analyses. This limitation is especially relevant to the distant-metastasis and later lymph-node-metastasis models, which included only 17 and 16 events, respectively. Although local recurrence was more frequent, with 41 events, its biopsy-derived effect estimate also remained relatively imprecise. Accordingly, the magnitude of the observed associations and the apparent incremental value of each specimen type should be interpreted cautiously and validated in larger independent cohorts. Finally, because biopsies inherently sample only a fraction of the invasive front, some degree of sampling error is unavoidable irrespective of the scoring methodology. Our section-level sensitivity analysis confirmed that the greater number of sections evaluated in resection specimens contributes materially to the higher detection of tumour budding. However, restricting resection assessment to a single section did not eliminate the difference relative to biopsy assessment, indicating that section number alone is insufficient to explain the discordance. Differences in tissue volume, anatomical sampling, representation of the deepest invasive front and spatial intratumoural heterogeneity are therefore likely to contribute additionally.

In conclusion, tumour budding represents a robust prognostic biomarker that is detectable in both biopsy and resection specimens. Resection-based assessment showed a numerically higher C-index for disease-specific survival and stronger evidence of association with occult and later nodal metastasis. Nevertheless, biopsy-derived tumour budding was associated with disease-specific survival, distant metastasis and local recurrence and retained prognostic information when considered alongside resection-based assessment, despite systematically underestimating high-risk disease. Given the small biopsy high-budding subgroup and the exploratory nature of the secondary endpoint analyses, these findings require validation in larger independent cohorts. Biopsy-derived tumour budding may therefore complement preoperative risk assessment but should not be considered a surrogate for comprehensive resection-based evaluation.

## Data Availability

The raw data supporting the conclusions of this article will be made available by the authors, without undue reservation.
